
JOURNAL INFORMATION
==============================
NLM Title Abbreviation: ScientificWorldJournal
Journal ID: ScientificWorldJournal
NLM Journal ID: 101131163
Journal ID: TSWJ

The Scientific World Journal

ISSN: 2356-6140
EISSN: 1537-744X
Publisher: Wiley

ARTICLE INFORMATION
==============================
PMCID: PMC6084059
PMID: 30147513
DOI: 10.1100/tsw.2001.185
Article ID: 305892
Article version: 1
Subjects: Short Report

Apoptosis of Spleenocytes and Expression of HIV Gene Products in the HIV-1 Transgenic Rat

Clark D.
Denaro F.J. *
Hayes N.
Jones O.
McCready M.
Davis H.
Reid W.
Bryant J.
Institute of Human Virology, Baltimore, MD, USA
*F.J. Denaro: denaro@umbi.umd.edu
Publication date: 2001
Electronic publication date: 2001 Dec 4
Volume: 1
First page: 52
Last page: 52
Copyright: Copyright © 2001 D. Clark et al.
Copyright year: 2001
License: This is an open access article distributed under the Creative Commons Attribution License, which permits unrestricted use, distribution, and reproduction in any medium, provided the original work is properly cited.
License URL: https://creativecommons.org/licenses/by/3.0/

Funding: Institute of Human Virology National Institutes of Health NS31857 National Institutes of Health MH29494

==============================
Miami *Nature Biotechnology* Short Reports
*TheScientificWorld* (2001) 1 (S3), 52SR
ISSN 1532-2246; DOI 10.1100/tsw.2001.185

### APOPTOSIS OF SPLEENOCYTES AND EXPRESSION OF HIV GENE PRODUCTS IN THE HIV-1 TRANSGENIC RAT

D. Clark, F.J. Denaro*, N. Hayes, O. Jones, M. McCready, H. Davis, W. Reid, and J. Bryant

Institute of Human Virology, Baltimore, MD
* Denaro@umbi.umd.edu

**INTRODUCTION.** Apoptosis is believed to play a major role in the progressive loss of CD4 + T cells, and other cell types in AIDS. Several studies support the theory that HIV-1 gene products may be the causative factor in the initiation of cell death (1-3). Moreover, post mortem studies reveal apoptotic cells in many tissues. Identification of mechanisms for the production of apoptosis in animal models of AIDS may clarify AIDS related cell death. A new model, developed at IHV for the study of AIDS related pathology is the HIV-1 Transgenic Rat. This model is unique among transgenic models in that measurable levels of Gp-120 have been identified in the serum and in spleenocyte lysates (4). In this study, we have characterized the location of HIV-1 transgene (GP120, NEF, TAT) expression in the spleen by means of immunocytochemistry. Moreover, identification of cells undergoing apoptosis was made in adjacent sections. The localization of HIV-1 gene products and the identification of apoptosis in this new animal model of AIDS could give insight in the mechanisms of HIV related cell lose.

**METHOD.** Paraffin sections of rat tissue were processed by standard immunohistochemical techniques using the ABC method (ABC Vector, Burlingame, CA). The HIV monoclonal antibodies that were used were anti- GP120, TAT and NEF. Additional sections were processed using the Apoptage (Integen). Tg rats were constructed by the pronuclei microinjection of the HIV-1-gag-pol- pEVd 1443 clone in which 3 kbps SphI and Bal1 fragment that contained the 3’ gag region and 5’ pol region of the infectious proviral DNA pNL4.3 clone was removed. This approach produced a non-infectious model (4).

**RESULTS.** Microscopic studies of the spleen with H&E stain revealed marked atypia of the splenic architecture. Evidence for cell loss as well has hyperplasia of the B-cell areas were noted (Fig. 1). Immunocytochemistry to GP-120 was identified in spleenocytes scattered through out the spleen (Fig. 3) and with a double label for macrophages and Gp-120 (Fig. 4). Negative controls were appropriate (Fig. 2). In addition, TAT and NEF were identified. Fig. 5 is an example of Nef labeling. Cells labeled with the apoptosis marker were found in different areas including both the T and B cell areas (Fig. 6).

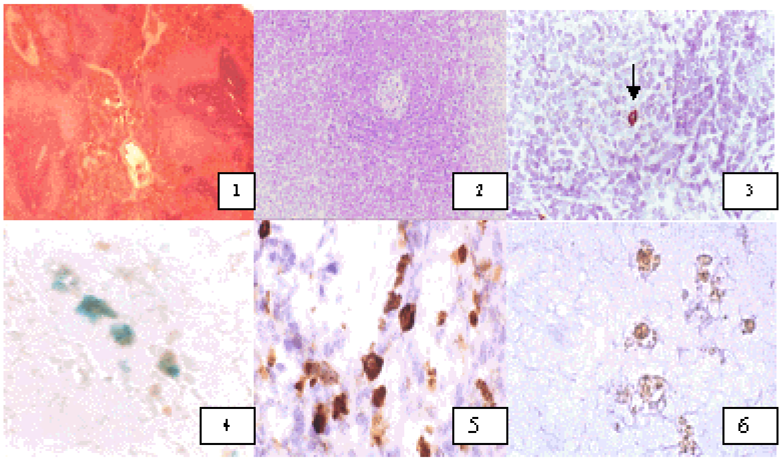

**DISCUSSION.** Using immunohistochemistry and labels for apoptosis, it was possible to identify the presence of HIV-1 transgenes (GP120, NEF, TAT) in the HIV-1 TGR spleen. Cell death of spleenocytes was identified in areas of transgene expression. These observations provide the bases for a new animal model of HIV infection in which HIV gene products and cell death can be studied.

**ACKNOWLEDGEMENT.** This work was supported by Institute of Human Virology, and by NIH grants NS31857 and MH29494.

**REFERENCES.**

1. Herbein, G., Van Lint, C., Lovett, J.L., and Verdin, E. (1998) J. Virol. 72, 660-670
2. Bartz, S.R. and Emerman, M. (1999) J. Virol. 73, 1956-1963
3. Azad, A.A. (2000) Biochem. Biophys. Res. Commun. 267, 677-685
4. Clark, D. et al. (2000) J. Hum. Virol. 3:5, 267
